# AI Denoising Significantly Improves Image Quality in Whole-Body Low-Dose Computed Tomography Staging

**DOI:** 10.3390/diagnostics12010225

**Published:** 2022-01-17

**Authors:** Andreas S. Brendlin, David Plajer, Maryanna Chaika, Robin Wrazidlo, Arne Estler, Ilias Tsiflikas, Christoph P. Artzner, Saif Afat, Malte N. Bongers

**Affiliations:** Department of Diagnostic and Interventional Radiology, Eberhard-Karls University, D-72076 Tuebingen, Germany; Andreas.Brendlin@med.uni-tuebingen.de (A.S.B.); David.Plajer@med.uni-tuebingen.de (D.P.); Maryanna.Chaika@med.uni-tuebingen.de (M.C.); Robin.Wrazidlo@med.uni-tuebingen.de (R.W.); Arne.Estler@med.uni-tuebingen.de (A.E.); Ilias.Tsiflikas@med.uni-tuebingen.de (I.T.); Christoph.Artzner@med.uni-tuebingen.de (C.P.A.); Malte.Bongers@med.uni-tuebingen.de (M.N.B.)

**Keywords:** computed tomography, tumor staging, AI (artificial intelligence), image quality enhancement, protection, radiation

## Abstract

(1) Background: To evaluate the effects of an AI-based denoising post-processing software solution in low-dose whole-body computer tomography (WBCT) stagings; (2) Methods: From 1 January 2019 to 1 January 2021, we retrospectively included biometrically matching melanoma patients with clinically indicated WBCT staging from two scanners. The scans were reconstructed using weighted filtered back-projection (wFBP) and Advanced Modeled Iterative Reconstruction strength 2 (ADMIRE 2) at 100% and simulated 50%, 40%, and 30% radiation doses. Each dataset was post-processed using a novel denoising software solution. Five blinded radiologists independently scored subjective image quality twice with 6 weeks between readings. Inter-rater agreement and intra-rater reliability were determined with an intraclass correlation coefficient (ICC). An adequately corrected mixed-effects analysis was used to compare objective and subjective image quality. Multiple linear regression measured the contribution of “Radiation Dose”, “Scanner”, “Mode”, “Rater”, and “Timepoint” to image quality. Consistent regions of interest (ROI) measured noise for objective image quality; (3) Results: With good–excellent inter-rater agreement and intra-rater reliability (Timepoint 1: ICC ≥ 0.82, 95% CI 0.74–0.88; Timepoint 2: ICC ≥ 0.86, 95% CI 0.80–0.91; Timepoint 1 vs. 2: ICC ≥ 0.84, 95% CI 0.78–0.90; all *p* ≤ 0.001), subjective image quality deteriorated significantly below 100% for wFBP and ADMIRE 2 but remained good–excellent for the post-processed images, regardless of input (*p* ≤ 0.002). In regression analysis, significant increases in subjective image quality were only observed for higher radiation doses (≥0.78, 95%CI 0.63–0.93; *p* < 0.001), as well as for the post-processed images (≥2.88, 95%CI 2.72–3.03, *p* < 0.001). All post-processed images had significantly lower image noise than their standard counterparts (*p* < 0.001), with no differences between the post-processed images themselves. (4) Conclusions: The investigated AI post-processing software solution produces diagnostic images as low as 30% of the initial radiation dose (3.13 ± 0.75 mSv), regardless of scanner type or reconstruction method. Therefore, it might help limit patient radiation exposure, especially in the setting of repeated whole-body staging examinations.

## 1. Introduction

Due to repeated follow-up examinations to monitor therapy, the most common indication for whole-body computed tomography (WBCT) is malignant diseases [1]. However, the substantial contribution of WBCT to the patients’ overall radiation exposure led to a growing concern in recent years regarding difficultly predictable long-term harms [2,3,4]. This concern is especially elevated in cancer patients, where studies show a significant rise in lifetime mortality from radiation-induced secondary malignancies [5,6]. Adjusting radiation dose exposure from radiological examinations “as low as reasonably achievable” (ALARA) has hence been the topic of a multitude of studies [7,8,9]. However, reducing the radiation dose in computed tomography is indivisibly linked to image quality deterioration due to rising image noise [10]. The limits of conventional reconstruction methods for image quality enhancements in low-dose computed tomography have previously been explored [11]. More recently, however, the advent of AI-based post-processing denoising solutions show promising results for further image quality enhancement [12,13,14]. However, as conventional reconstruction methods, novel AI-based techniques have specific characteristics and caveats essential to consider, such as reduced spatial information, blurring, and possible loss of information [15]. Therefore, recent review articles have pointed out the necessity to research the utility of such solutions on a use case level [16,17]. In the setting of metastatic melanoma, organ metastases are an essential determinant for overall survival, regardless of primary tumor location [18]. However, facilitating low-dose WBCT for patients with metastatic melanoma is no easy task, as rising image noise can severely complicate proper visual assessment [19]. This study aimed to evaluate the effects of an AI denoising algorithm on image quality in WBCT stagings of melanoma patients (ocular and cutaneous). We hypothesize that the software may produce diagnostic images at low radiation doses beyond the limits of conventional reconstruction methods and thus help limit radiation exposure.

## 2. Materials and Methods

### 2.1. Study Design, Population, and Radiation Dose

The institutional review board approved retrospective image data collection for this single-center study’s purpose with a waiver for the need for informed consent (#414/2017BO2). Therefore, from 1 January 2019 to 1 January 2021, we retrospectively included melanoma patients with clinically indicated WBCT staging from two scanners from our clinical routine. First, we collected the patients’ age, sex, height, and weight and computed their body mass index (BMI in kg/m^2^). Next, we selected 60 patients per scanner from the initial patient inclusion with exactly matching biometric profiles (same age, same sex, same BMI). Then, from the dose reports of the WBCT, we collected the Computer Tomography Dose Index (CTDI_vol_ in mGy) and the dose-length product (DLP in mGy × cm) and computed the effective radiation dose (ED in mSv) using appropriate weighting factors [20].

### 2.2. Image Acquisition and Reconstruction Parameters

We used CT examinations from two CT scanners for this study: SOMATOM Definition AS+ and SOMATOM Force (Siemens Healthineers, Erlangen, Germany). Both scanners employed attenuation-based tube current modulation (CARE Dose4D, reference mAs 190) and automatic tube voltage selection (80–120 kV, reference kV 110). On SOMATOM Definition AS+, collimation was set to 0.6 × 64 mm, and on SOMATOM Force to 0.6 × 96 mm. Pitch was 0.6, gantry rotation time was 0.5 s, and matrix size was 512 for both CT scanners. For the WBCT stagings, the patients were positioned head-first on their back with elevated arms. All analyzed scans were contrast-enhanced using Imerone 400 (Bracco, Milan, Italy). An automated power injector applied the contrast medium through a peripheral venous cannula at a flow rate of 2.2 ± 0.5 mL/s (CT Stellant, Medrad, Indianola, PA, USA) followed by a chaser of 50 mL saline. Images were acquired in a portal venous phase at 80–90 s after administration of contrast medium. The WBCT images from both scanners were reconstructed with equivalent medium-soft kernels (Br36f for SOMATOM Definition AS+ and Bf40d for SOMATOM Force) in axial orientation with a slice thickness and an increment of 1 mm. We used two conventional reconstruction methods (weighted filtered back-projection (wFBP) and Advanced Modeled Iterative Reconstruction strength 2 (ADMIRE^®^, Siemens Healthineers, Erlangen, Germany)) for image reconstruction. All reconstructions were performed offline using a dedicated software solution (ReconCT ver. 14.2.0.4998, Siemens Healthineers, Erlangen, Germany) that allows for retrospective noise insertion to simulate acquisition at lower tube currents (mAs). In addition to full radiation dose reference datasets (100% mAs), we thus simulated 50%, 40%, and 30% radiation dose. Furthermore, a novel AI-based post-processing software solution (PixelShine^®^, AlgoMedica, Sunnyvale, CA, USA) was used to denoise all WBCT images, resulting in four datasets per radiation dose level and 16 datasets per examination.

### 2.3. Image Quality Analysis

#### 2.3.1. Subjective Image Quality

The patient datasets were anonymized and randomized by a group member otherwise not associated with subjective image quality analysis. Five readers with different experience levels in WBCT staging independently rated subjective image quality on a 5-point Likert scale (1 = poor, 2 = subpar, 3 = fair, 4 = good, 5 = excellent) according to the diagnostic requirements mentioned in the chapters “Chest, General” and “Abdomen, General” of the European Guidelines on Image Quality in Computed Tomography [21]. Each reader rated the datasets two times with six weeks between each session.

#### 2.3.2. Objective Image Quality

Objective image quality analysis was performed in MatLab (Ver. R2021a, The MathWorks, Natick, MA, USA), using a previously described, custom-built script [22]. This script allows for consistent region of interest (ROI) measurements across matching sets of examinations. We placed 6 ROI in homogenous areas of paraspinal muscles in 5 consecutive slices. The MatLab script automatically extracted mean CT numbers in Hounsfield units (HU) and their standard deviations (SD) per ROI. The SD of HU was defined as image noise and used to measure objective image quality. 

### 2.4. Statistical Analysis

Statistical analysis and illustration were performed using GraphPad Prism version 9.3 for Windows (GraphPad Software, San Diego, CA, USA). Data distribution was tested using the Shapiro–Wilk test. Normally distributed variables were expressed as mean ± SD, and non-normally distributed variables as median and interquartile range (IQR). Data analysis ensued using a mixed-effects model with Greenhouse–Geisser correction in case of violation of sphericity. In addition, Bonferroni correction was used for multiple comparisons to counteract type 1 error increase. An adjusted *p*-value ≤ 0.05 indicated statistical significance. Multiple linear regression with three-way interactions was utilized to investigate the contribution of the variables “Effective Radiation Dose” (ED in mSv, reference category 30%), “Scanner” (CT scanner, reference category SOMATOM Definition AS+), “Mode” (reconstruction/post-processing mode, reference category wFBP), “Rater” (reference category Rater 1), and “Timepoint” (first/second subjective rating, reference category timepoint 1) to subjective image quality. The utility and goodness-of-fit of the multiple linear regression model were measured using analysis of variance (ANOVA), adjusted R^2^, and the standard deviation of the residuals (Sy.x). R^2^ values of ≤0.13 were considered indicative for poor, 0.13–0.26 for moderate, and ≥0.26 for high goodness-of-fit [23]. To quantify the subjective image quality scores’ inter-rater agreement and intra-rater variability, we used an intraclass correlation coefficient (ICC, two-way mixed, absolute agreement, average measures) with 95% confidence intervals (95%CI) [24]. ICC values of 0–0.5 were considered poor, 0.51–0.74 moderate, 0.75–0.9 good, and 0.91–1.00 excellent levels of agreement. 

## 3. Results

### 3.1. Study Population and Radiation Dose

**Figure 1:**
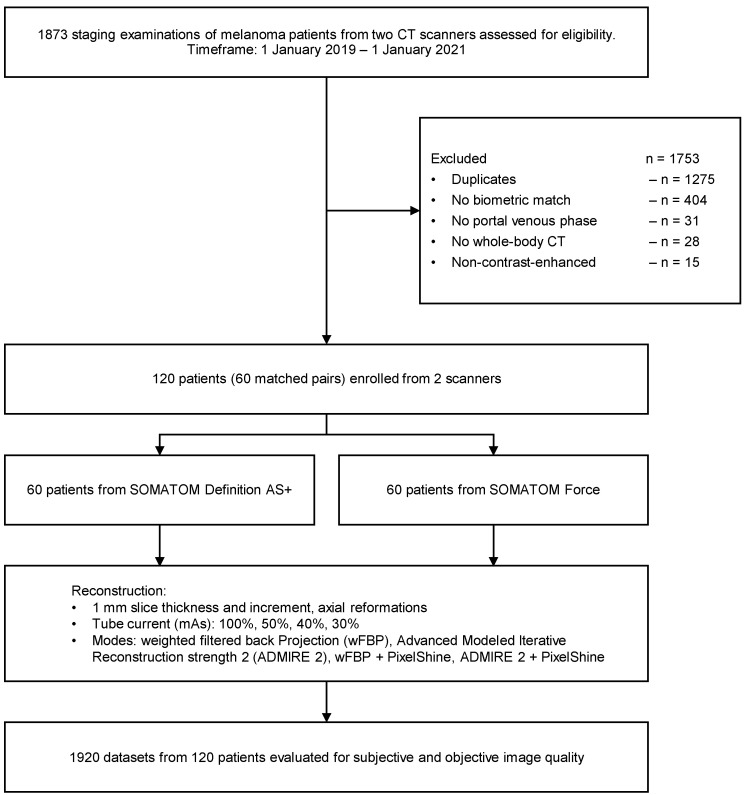
Study flowchart and patient enrollment. wFBP = weighted filtered back-projection; ADMIRE 2 = Advanced Modeled Iterative Reconstruction strength 2.

The initial database search (keywords: “melanoma staging”) revealed a total of 1873 melanoma patients (ocular and cutaneous) with clinically indicated CT staging from 1 January 2019 to 1 January 2021 on two scanners (SOMATOM Definition AS+, SOMATOM Force) for eligibility assessment. If patients had more than one WBCT in the given timeframe, only the first scan was included, and the others (duplicates) were excluded. We selected all patients with exactly matching biometric profiles (same age, same sex, same BMI) and excluded all patients without exact match. Further exclusion criteria were no portal venous phase, no whole-body CT, and non-contrast-enhanced examinations. Thus, 1753 patients were excluded, and 120 patients were enrolled in the study (60 patients per scanner). For details about our study population (see Table 1). Figure 1 visualizes the study workflow and the patient enrollment.

### 3.2. Image Quality Analysis

#### 3.2.1. Subjective Image Quality

On SOMATOM Definition AS+, overall subjective image quality at 100% radiation dose was good (4 (4–4)) for wFBP and good (4 (4–4)) for ADMIRE 2. These ratings decreased to poor at 30% radiation dose (1 (1–2)) for both reconstruction modes. The post-processed images had good image quality (4 (4–5)) at each radiation dose. On SOMATOM Force, overall subjective image quality at 100% radiation dose was good (4 (4–5)) for wFBP and excellent (5 (4–5)) for ADMIRE 2. At 30% radiation dose, these ratings decreased to poor (1 (1–2)) for wFBP and subpar (2 (1–2)) for ADMIRE 2. At 100%, both post-processed images from both reconstruction modes were rated excellent (≥5 (4–5)), decreasing to good (4 (4–5)) image quality at lower radiation doses. Regardless of CT scanner, radiation dose, or reconstruction mode, mixed-effects analysis with pairwise comparisons showed no significant differences between the subjective image quality ratings of all post-processed images (*p* ≥ 0.069), as well as between the post-processed images and the 100% wFBP and ADMIRE 2 images (*p* ≥ 0.245). While the subjective image quality ratings overall deteriorated significantly for decreasing radiation dose (*p* ≤ 0.002), there were no significant differences between wFBP and ADMIRE 2 at each level (*p* ≥ 0.611). The inter-rater agreement and the intra-rater reliability of the subjective image quality ratings were good to excellent (Timepoint 1: ICC ≥ 0.82, 95% CI 0.74–0.88; Timepoint 2: ICC ≥ 0.86, 95% CI 0.80–0.91; Timepoint 1 vs. 2: ICC ≥ 0.84, 95% CI 0.78–0.90; all *p* ≤ 0.001). Table 2 shows further details about the ratings for each timepoint, the inter-rater agreement, and the intra-rater reliability.

**Figure 2:**
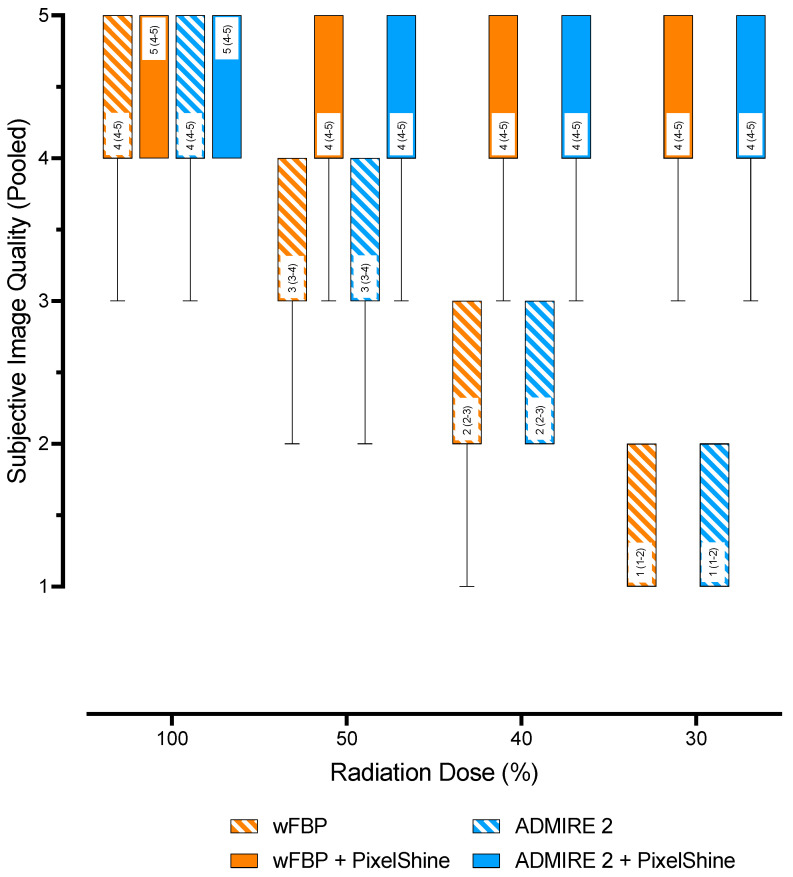
Pooled subjective image quality scores. wFBP = weighted Filtered Back Projection, ADMIRE 2 = Advanced Modeled Iterative Reconstruction strength 2.

The multiple linear regression model was able to predict subjective image quality (F (178; 19 021) = 364; *p* < 0.001) and showed a high goodness-of-fit (adjusted R^2^ = 0.77, Sy.x = 0.62). The variables “Scanner: SOMATOM Force”, “Mode: Admire 2”, “Rater: Rater 2–5”, and “Timepoint: Timepoint 2” did not contribute to increases in subjective image quality (*p* > 0.564). Significant increases in subjective image quality were observed for higher radiation doses (≥0.78, 95%CI 0.63–0.93 points; *p* < 0.001), as well as for “Mode: ADMIRE 2 + PS” (2.88, 95%CI 2.72–3.03 points, *p* < 0.001) and “Mode: wFBP + PS” (3.06, 95%CI 2.91–3.21 points, *p* < 0.001). Table 3 shows additional regression metrics. Figure 2 shows pooled subjective image quality ratings (all raters, all timepoints, all scanners) as a function of “Effective Radiation Dose” and “Mode”.

**Figure 3:**
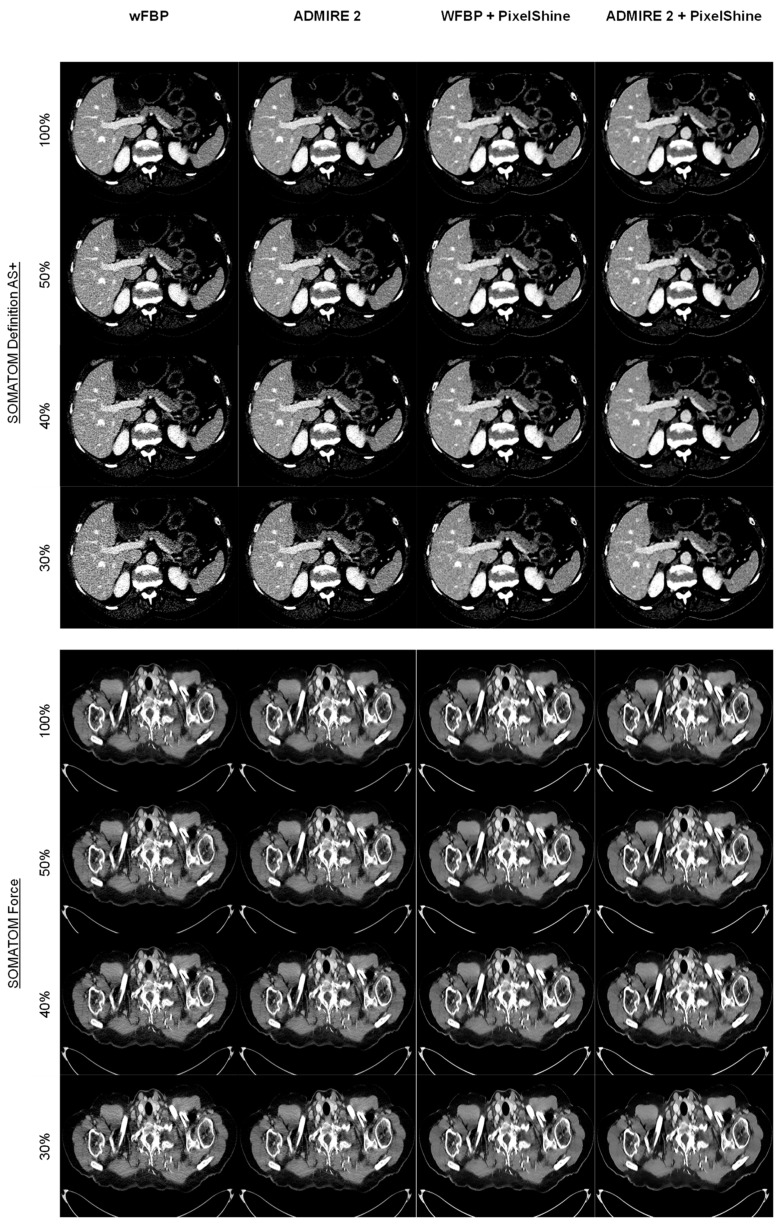
Comparison of image quality for all scanner types, radiation dose levels, and reconstruction methods. wFBP = weighted Filtered Back Projection, ADMIRE 2 = Advanced Modeled Iterative Reconstruction strength 2.

Figure 3 shows comparison images for both scanners across all radiation dose levels and reconstruction modes. Of note is the consistent image quality of the post-processed images when compared to conventional methods, with only minor decreases in image sharpness below 100%. 

#### 3.2.2. Objective Image Quality

**Figure 4:**
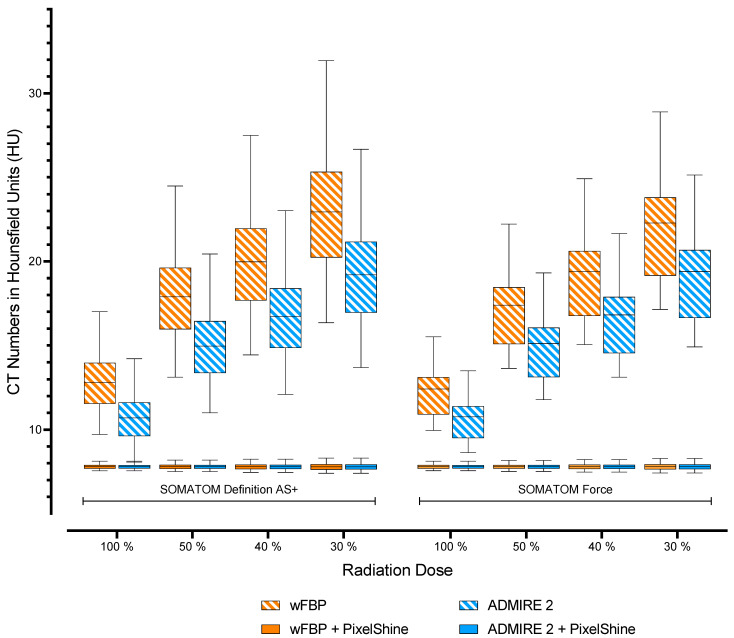
Visualization of mean noise levels. wFBP = weighted Filtered Back Projection, ADMIRE 2 = Advanced Modeled Iterative Reconstruction strength 2.

For both scanners, wFBP reconstructions had significantly higher image noise than ADMIRE 2 reconstructions at each radiation dose level (*p* < 0.001). Nevertheless, direct comparisons of image noise from wFBP and ADMIRE 2 reconstructions between SOMATOM AS+ and SOMATOM Force showed no significant differences (*p* ≥ 0.987). Furthermore, all post-processed images had significantly lower image noise than the standard wFBP and ADMIRE 2 reconstructions (*p* < 0.001), with no differences between the post-processed images themselves, regardless of scanner type, radiation dose, or reconstruction mode (*p* ≥ 0.255). Table 4 shows mean image noise values of all datasets with pairwise comparisons between each scanner group (SOMATOM Definition AS+ vs. SOMATOM Definition AS+, SOMATOM Force vs. SOMATOM Force). Figure 4 visualizes the measured noise levels. 

**Figure 5:**
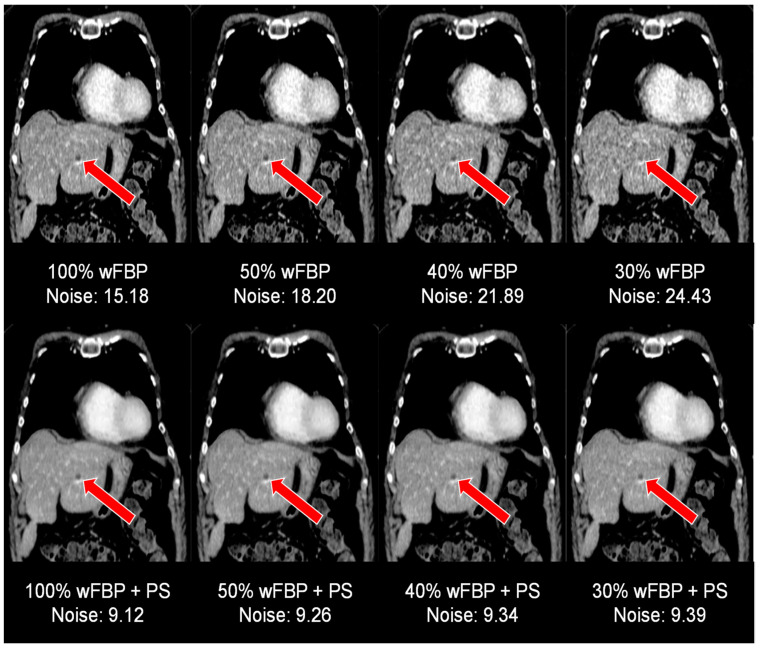
Image quality aspects in the setting of a hepatic melanoma metastasis at the different radiation dose levels, conventional reconstruction (top row) vs. postprocessing (bottom row). wFBP = weighted Filtered Back Projection, PS = PixelShine.

Figure 5 visualizes image quality aspects in the setting of a hepatic melanoma metastasis (marked with red arrows) in a 54-year-old woman at different radiation dose levels using conventional reconstruction methods (top row) and post-processing (bottom row). Note the highly enhanced image quality in the post-processed images, facilitating diagnostic assessment as low as 30% of the initial radiation dose. 

## 4. Discussion

In CT imaging, radiation dose reduction is vital to promote patient safety and minimize the risk for difficultly predictable long-term harms, especially for secondary malignancies. However, radiation dose reduction is indivisibly linked with image quality deterioration due to increasing image noise. Thus, balancing safety versus image quality can be difficult, especially in cancer patients who need repeated follow-up whole-body CT scans. This study evaluated an AI-based post-processing denoising software solution regarding image quality compared to conventional reconstruction methods. Regardless of input radiation dose or scanner type, the software offered a significantly larger dose reduction potential than wFBP and ADMIRE reconstruction. In our study, subjective image quality analysis confirmed decreases at lower radiation doses for conventional reconstruction methods but showed high subjective image quality for the post-processed images. These results are in line with previous studies. Shin et al. reported excellent image quality at 50% radiation dose without significant differences to their 100% reference ADMIRE reconstruction [25]. Converting their results into effective radiation dose, they measured lower absolute dose levels at 50% in comparison with what we did at 30% in our study. However, it is worth pointing out that they investigated abdominal CT scans instead of whole-body scans. Although there was no statistical significance in the decrease from excellent to good image quality on SOMATOM Force, it is still noteworthy that there was a slight drop in image quality from 100% to 50%. In the post hoc unblinded results discussion, our readers pointed out this might have mostly been due to slightly decreasing image sharpness. As image sharpness was already part of our subjective image quality assessment criteria, we did not further investigate this effect. Previous studies have nonetheless described similar results. Shin et al. reported a significant loss of spatial resolution at radiation doses below 50% [25]. Furthermore, Kang et al. indicated a significant blurring effect that may be introduced by denoising [26]. However, it is noteworthy that our setup used a newer CT scanner generation than both these studies. Therefore, we hypothesize this effect to be more prevalent in older scanner generations. As expected, multiple linear regression showed significant image quality increases for rising radiation doses. Interestingly, the model showed that the different scanners used in this study, and the conventional reconstruction modes did not significantly increase subjective image quality. A significant contribution to image quality was observed for the post-processing algorithm, with the highest estimate for wFBP + PixelShine. Previous studies have described similar results with higher image quality enhancement potentials for wFBP than ADMIRE reconstructions. Hata et al., for example, described relatively smaller image quality improvements for model-based iterative reconstruction input images than for wFBP images when using denoising algorithms [27]. In conjunction with the results of previous studies, they argued wFBP images have a greater room for improvement than iteratively reconstructed images [28]. Looking at the multiple linear regression estimates in synopsis with our study’s subjective image quality analysis scores, we found that ADMIRE reconstructions predominantly received higher scores than their wFBP counterparts. Therefore, we conclude that this result is due to the relational nature of multiple linear regression itself. As expected, in objective image quality analysis, we measured significantly lower noise levels for the post-processed datasets than for conventional reconstruction methods. It is, however, essential to reinforce the fact that these results were stable, regardless of scanner type or radiation dose. Especially in the setting of repeated CT examinations to monitor tumor treatment, the investigated algorithm can contribute significantly to radiation dose reduction and thus potentially decrease the risk of secondary malignancies. In our study, the investigated algorithm facilitated significantly reduced radiation doses in the setting of repeated WBCT staging examinations and, therefore, potentially decreases the risk of secondary malignancies. This study has several limitations. First, this was a retrospective study with 120 patients. Although a total of 1920 datasets were reviewed, a prospective follow-up study is merited to confirm the implications of our results for clinical decision-making. Second, this study used biometrically matching patient cohorts from two scanners and employed realistic low-dose simulations to prevent repeated radiation exposure. If feasible, the power of similar future studies could further benefit from prospective low-dose CT acquisition in an intraindividual setting. Third, multiple studies have pointed out unfamiliar appearances, loss of spatial information, and blurring in AI denoising post-processing. Therefore, it might be best to handle the generalizability of the results of such algorithms with caution and reevaluate them for specific medical questions on a use case level. Fourth, although performed in an oncological setting, this study focused on image quality aspects of overall organ visibility rather than specific tumor staging. Further studies will be needed to confirm our results regarding lesion detectability in denoised low dose CT datasets. Lastly, this study utilized two CT scanners from one vendor, which might not be readily available at every site. Our results might therefore be specific to this setup.

## 5. Conclusions

The investigated AI post-processing software solution produces diagnostic images as low as 30% of the initial radiation dose (3.13 ± 0.75 mSv), regardless of scanner type or reconstruction method. Therefore, it might help limit patient radiation exposure, especially in the setting of repeated whole-body staging examinations.

## Figures and Tables

**Table 1:** Patient characteristics and radiation dose.

				Female	Male	Overall
SOMATOM Definition AS+	Patient characteristics					
		N		30	30	60
		Age		63 ± 12	59 ± 16	61 ± 14
		BMI		26 ± 3	27 ± 2	26 ± 3
	Radiation dose					
		CTDI_vol_ (mean ± SD)		4.31 ± 0.95	4.46 ± 0.94	4.38 ± 0.95
		DLP (mean ± SD)		746.63 ± 165.58	759.54 ± 168.45	753.08 ± 167.15
		ED (mean ± SD)				
			100% mAs	10.72 ± 2.53	11.39 ± 2.53	11.05 ± 2.55
			50% mAs	5.36 ± 1.27	5.70 ± 1.26	5.53 ± 1.28
			40% mAs	4.29 ± 1.01	4.56 ± 1.01	4.43 ± 1.02
			30% mAs	3.22 ± 0.76	3.42 ± 0.76	3.32 ± 0.77
SOMATOM Force	Patient characteristics					
		N		30	30	60
		Age		63 ± 12	59 ± 16	61 ± 14
		BMI		26 ± 3	27 ± 2	26 ± 3
	Radiation dose					
		CTDI_vol_ (mean ± SD)		4.15 ± 0.97	4.32 ± 0.97	4.22 ± 0.97
		DLP (mean ± SD)		714.42 ± 168.85	726.78 ± 171.77	720.60 ± 170.43
		ED (mean ± SD)				
			100% mAs	9.17 ± 2.17	9.59 ± 2.13	9.38 ± 2.16
			50% mAs	4.59 ± 1.08	4.79 ± 1.06	4.69 ± 1.08
			40% mAs	3.67 ± 0.87	3.83 ± 0.85	3.75 ± 0.86
			30% mAs	2.75 ± 0.65	2.88 ± 0.64	2.81 ± 0.65

N = number; SD = standard deviation; BMI = body mass index (kg/m^2^); CTDI_vol_ = Computer Tomography Dose Index (mGy); DLP = dose length product (mGy x cm); ED = effective radiation dose (mSv).

**Table 2:** Subjective image quality ratings, inter-rater-agreement, and intra-rater reliability.

			Timepoint 1			Timepoint 1			Timepoint 1 vs. 2	
			Rating	Inter-Rater Agreement		Rating	Inter-Rater Agreement		Intra-Rater Reliability	
	ED	Mode	Median (IQR)	ICC (95% CI)	*p*	Median (IQR)	ICC (95% CI)	*p*	ICC (95% CI)	*p*
SOMATOM Definition AS+	100%	wFBP	4 (4–4)	0.97 (0.96–0.98)	<0.001	4 (3–4)	0.95 (0.93–0.97)	<0.001	0.98 (0.97–0.99)	<0.001
		ADMIRE 2	4 (4–5)	0.96 (0.94–0.98)	<0.001	4 (4–5)	0.96 (0.95–0.98)	<0.001	0.98 (0.97–0.98)	<0.001
		wFBP + PS	4 (4–5)	0.97 (0.96–0.98)	<0.001	4 (4–5)	0.86 (0.80–0.91)	<0.001	0.98 (0.97–0.98)	<0.001
		ADMIRE 2 + PS	4 (4–5)	0.97 (0.95–0.98)	<0.001	4 (4–5)	0.93 (0.90–0.96)	<0.001	0.98 (0.98–0.99)	<0.001
	50%	wFBP	3 (3–3)	0.98 (0.98–0.99)	<0.001	3 (2–3)	0.98 (0.97–0.99)	<0.001	0.98 (0.98–0.99)	<0.001
		ADMIRE 2	3 (3–4)	0.98 (0.97–0.99)	<0.001	3 (3–4)	0.97 (0.95–0.98)	<0.001	0.98 (0.97–0.99)	<0.001
		wFBP + PS	4 (4–5)	0.94 (0.92–0.96)	<0.001	4 (4–5)	0.89 (0.83–0.93)	<0.001	0.94 (0.92–0.96)	<0.001
		ADMIRE 2 + PS	4 (4–5)	0.95 (0.93–0.97)	<0.001	4 (4–5)	0.93 (0.90–0.96)	<0.001	0.95 (0.93–0.97)	<0.001
	40%	wFBP	2 (2–2)	0.98 (0.97–0.99)	<0.001	2 (1–2)	0.97 (0.95–0.98)	<0.001	0.99 (0.98–0.99)	<0.001
		ADMIRE 2	2 (2–2)	0.99 (0.98–0.99)	<0.001	2 (2–3)	0.96 (0.94–0.97)	<0.001	0.98 (0.97–0.99)	<0.001
		wFBP + PS	4 (4–5)	0.92 (0.89–0.95)	<0.001	4 (4–5)	0.91 (0.87–0.94)	<0.001	0.92 (0.88–0.95)	<0.001
		ADMIRE 2 + PS	4 (4–5)	0.95 (0.93–0.97)	<0.001	4 (4–5)	0.95 (0.92–0.96)	<0.001	0.95 (0.92–0.96)	<0.001
	30%	wFBP	1 (1–1)	0.99 (0.98–0.99)	<0.001	1 (1-1)	0.96 (0.94–0.97)	<0.001	0.98 (0.97–0.99)	<0.001
		ADMIRE 2	1 (1–2)	0.97 (0.96–0.98)	<0.001	1 (1-2)	0.96 (0.94–0.97)	<0.001	0.98 (0.97–0.99)	<0.001
		wFBP + PS	4 (4–5)	0.91 (0.87–0.94)	<0.001	4 (4-5)	0.96 (0.94–0.98)	<0.001	0.91 (0.87–0.94)	<0.001
		ADMIRE 2 + PS	4 (4–5)	0.95 (0.92–0.96)	<0.001	4 (4-5)	0.98 (0.97–0.98)	<0.001	0.94 (0.92–0.96)	<0.001
SOMATOM Force	100%	wFBP	4 (4–5)	0.98 (0.97–0.99)	<0.001	4 (4–5)	0.98 (0.97–0.99)	<0.001	0.99 (0.98–0.99)	<0.001
		ADMIRE 2	5 (4–5)	0.95 (0.92–0.97)	<0.001	5 (4–5)	0.96 (0.95–0.98)	<0.001	0.96 (0.95–0.98)	<0.001
		wFBP + PS	5 (4–5)	0.82 (0.74–0.88)	<0.001	5 (4–5)	0.89 (0.84–0.93)	<0.001	0.84 (0.78–0.90)	<0.001
		ADMIRE 2 + PS	5 (5–5)	0.91 (0.86–0.94)	<0.001	5 (4–5)	0.91 (0.87–0.94)	<0.001	0.85 (0.79–0.90)	<0.001
	50%	wFBP	3 (3–4)	0.99 (0.98–0.99)	<0.001	3 (3–4)	0.98 (0.97–0.99)	<0.001	0.99 (0.98–0.99)	<0.001
		ADMIRE 2	4 (3–4)	0.96 (0.94–0.97)	<0.001	4 (3–4)	0.95 (0.93–0.97)	<0.001	0.97 (0.96–0.98)	<0.001
		wFBP + PS	4 (4–5)	0.92 (0.88–0.95)	<0.001	4 (4–5)	0.91 (0.88–0.94)	<0.001	0.84 (0.77–0.89)	<0.001
		ADMIRE 2 + PS	4 (4–5)	0.94 (0.92–0.96)	<0.001	4 (4–5)	0.83 (0.75–0.89)	<0.001	0.88 (0.83–0.92)	<0.001
	40%	wFBP	2 (2–3)	0.99 (0.99–1.00)	<0.001	2 (2–3)	0.98 (0.96–0.98)	<0.001	0.99 (0.98–0.99)	<0.001
		ADMIRE 2	3 (2–3)	0.98 (0.97–0.99)	<0.001	3 (2–3)	0.95 (0.92–0.97)	<0.001	0.97 (0.96–0.98)	<0.001
		wFBP + PS	4 (4–5)	0.92 (0.88–0.94)	<0.001	4 (4–5)	0.86 (0.80–0.91)	<0.001	0.86 (0.80–0.91)	<0.001
		ADMIRE 2 + PS	4 (4–5)	0.93 (0.89–0.95)	<0.001	4 (4–5)	0.9 (0.85–0.93)	<0.001	0.85 (0.79–0.90)	<0.001
	30%	wFBP	1 (1–2)	0.98 (0.97–0.99)	<0.001	1 (1–2)	0.97 (0.96–0.98)	<0.001	0.99 (0.99–0.99)	<0.001
		ADMIRE 2	2 (1–2)	0.96 (0.94–0.97)	<0.001	2 (1–2)	0.93 (0.89–0.95)	<0.001	0.98 (0.97–0.99)	<0.001
		wFBP + PS	4 (4–5)	0.86 (0.80–0.91)	<0.001	4 (4–5)	0.92 (0.88–0.95)	<0.001	0.87 (0.82–0.91)	<0.001
		ADMIRE 2 + PS	4 (4–5)	0.92 (0.88–0.95)	<0.001	4 (4–5)	0.95 (0.92–0.96)	<0.001	0.93 (0.90–0.95)	<0.001

ED = effective radiation dose; wFBP = weighted filtered back-projection; ADMIRE 2 = Advanced Modeled Iterative Reconstruction strength 2; PS = PixelShine; ICC = intraclass correlation coefficient; 95% CI = 95% confidence interval; *p* = significance level.

**Table 3:** Linear regression metrics: subjective image quality.

Variable	B	SE	95% CI	|t|	*p*
Intercept	1.21	0.06	1.09–1.33	20.1	<0.001
ED (reference: 30%)					
40%	0.78	0.08	0.63–0.93	10.1	<0.001
50%	1.78	0.08	1.63–1.93	23	<0.001
100%	2.78	0.08	2.63–2.93	35.9	<0.001
Scanner (reference: SOMATOM Definition AS+)					
SOMATOM Force	0.22	0.07	0.09–0.35	3.26	0.053
Mode (reference: wFBP)					
ADMIRE 2	0.04	0.08	−0.11–0.2	0.58	0.564
ADMIRE 2 + PS	2.88	0.08	2.72–3.03	37.2	<0.001
wFBP + PS	3.06	0.08	2.91–3.21	39.6	<0.001
Rater (reference: Rater 1)					
Rater 2	0.01	0.08	–0.16–0.16	0	>0.999
Rater 3	0.01	0.08	–0.16–0.16	0	>0.999
Rater 4	0.01	0.08	–0.14–0.17	0.15	0.882
Rater 5	0.01	0.08	–0.16–0.16	0	>0.999
Timepoint (reference: Timepoint 1)					
Timepoint 2	0.03	0.07	–0.1–0.16	0.46	0.648

B = estimate; SE = standard error; 95% CI = 95% confidence interval; |t| = absolute value of t statistics, *p* = significance level; ED = effective radiation dose; Mode = reconstruction/postprocessing mode; wFBP = weighted filtered back-projection; ADMIRE 2 = Advanced Modeled Iterative Reconstruction strength 2; PS = PixelShine; Timepoint = time of subjective image quality rating.

**Table 4:** Mean noise levels and pairwise noise comparisons.

			Noise	*p* (Two-Sided, Adjusted) vs. 100% ED			
	ED	Mode	Mean ± SD	wFBP	ADMIRE 2	wFBP + PS	ADMIRE 2 + PS
SOMATOM Definition AS+	100%	wFBP	12.85 ± 1.63		<0.001	<0.001	<0.001
		ADMIRE 2	10.75 ± 1.36	<0.001		<0.001	<0.001
		wFBP + PS	7.8 ± 0.13	<0.001	<0.001		0.423
		ADMIRE 2 + PS	7.8 ± 0.13	<0.001	<0.001	0.423	
	50%	wFBP	17.99 ± 2.51	<0.001	<0.001	<0.001	<0.001
		ADMIRE 2	15.05 ± 2.11	<0.001	<0.001	<0.001	<0.001
		wFBP + PS	7.8 ± 0.15	<0.001	<0.001	0.936	0.943
		ADMIRE 2 + PS	7.8 ± 0.15	<0.001	<0.001	0.860	0.991
	40%	wFBP	20.06 ± 2.89	<0.001	<0.001	<0.001	<0.001
		ADMIRE 2	16.78 ± 2.42	<0.001	<0.001	<0.001	<0.001
		wFBP + PS	7.8 ± 0.18	<0.001	<0.001	0.947	0.941
		ADMIRE 2 + PS	7.8 ± 0.17	<0.001	<0.001	0.963	0.969
	30%	wFBP	23.07 ± 3.46	<0.001	<0.001	<0.001	<0.001
		ADMIRE 2	19.29 ± 2.89	<0.001	<0.001	<0.001	<0.001
		wFBP + PS	7.8 ± 0.2	<0.001	<0.001	0.980	0.981
		ADMIRE 2 + PS	7.8 ± 0.2	<0.001	<0.001	0.979	0.969
SOMATOM Force	100%	wFBP	12.35 ± 1.57	<0.001	<0.001	<0.001	<0.001
		ADMIRE 2	10.73 ± 1.36	<0.001		<0.001	<0.001
		wFBP + PS	7.8 ± 0.13	<0.001	<0.001		0.422
		ADMIRE 2 + PS	7.8 ± 0.12	<0.001	<0.001	0.422	
	50%	wFBP	17.29 ± 2.42	<0.001	<0.001	<0.001	<0.001
		ADMIRE 2	15.02 ± 2.11	<0.001	<0.001	<0.001	<0.001
		wFBP + PS	7.8 ± 0.15	<0.001	<0.001	0.927	0.807
		ADMIRE 2 + PS	7.8 ± 0.14	<0.001	<0.001	0.920	0.927
	40%	wFBP	19.27 ± 2.78	<0.001	<0.001	<0.001	<0.001
		ADMIRE 2	16.74 ± 2.41	<0.001	<0.001	<0.001	<0.001
		wFBP + PS	7.8 ± 0.17	<0.001	<0.001	0.930	0.868
		ADMIRE 2 + PS	7.8 ± 0.17	<0.001	<0.001	0.936	0.895
	30%	wFBP	22.17 ± 3.32	<0.001	<0.001	<0.001	<0.001
		ADMIRE 2	19.26 ± 2.89	<0.001	<0.001	<0.001	<0.001
		wFBP + PS	7.8 ± 0.19	<0.001	<0.001	0.936	0.897
		ADMIRE 2 + PS	7.8 ± 0.19	<0.001	<0.001	0.978	0.978

ED = effective radiation dose; SD = standard deviation; mAs = tube current; wFBP = weighted Filtered Back Projection; ADMIRE 2 = Advanced Modeled Iterative Reconstruction strength 2; PS = PixelShine.

## Data Availability

Data is contained within the article.
